# Duodenal endoscopic submucosal dissection for a fibrotic recurrent adenoma using combined undersaline immersion, traction, and hook-drag-cut dissection techniques

**DOI:** 10.1055/a-2877-2338

**Published:** 2026-06-23

**Authors:** Georgios Mavrogenis, Michalis Tsakoniatis, Dimitrios Madianos

**Affiliations:** 1 Third Space Endoscopy Unit 168211 Mediterraneo HospitalAthensGreece

Endoscopic submucosal dissection (ESD) in the duodenum is widely considered the most challenging ESD procedure and should be performed only in high-volume centers due to the thin wall and substantial risk of perforation, particularly in the presence of fibrosis following prior interventions. 1​ ​2​ We present a combined strategy using the undersaline immersion technique, countertraction and the hook-drug-cut dissection technique for the safe resection of a recurrent non-ampullary duodenal adenoma ( Video 1 ).

**Video 1** Video demonstration of the undersaline immersion technique, countertraction and hook-drug-cut dissection technique for a recurrent duodenal adenoma.


**Fig. 1:**
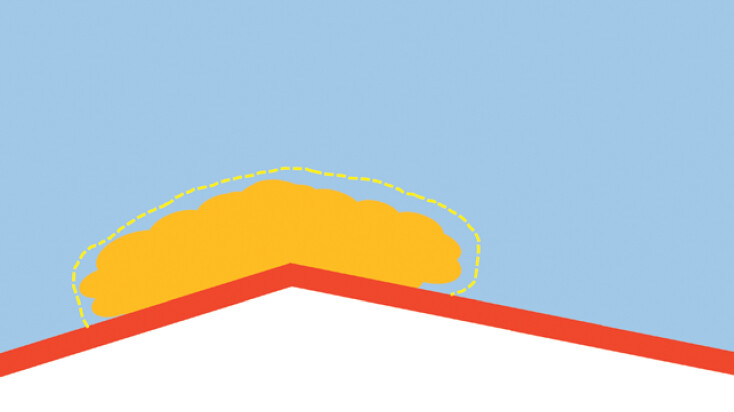
The lumen was filled with saline to achieve undersaline immersion, followed by circumferential mucosal incision.

A 70-year-old woman was referred to our center with a 1.5-cm recurrent adenoma located in the second portion of the duodenum after multiple previous polypectomy attempts. Given the significant fibrosis, ESD was selected and performed under general anesthesia with a surgical backup. The lumen was filled with normal saline to create an undersaline environment, and lesion margins were carefully delineated using a needle-type knife in a forced coagulation mode. A circumferential mucosal incision was followed by the creation of a lateral mucosal flap to facilitate access to the submucosal space ( Fig. 1 ).

**Fig. 2:**
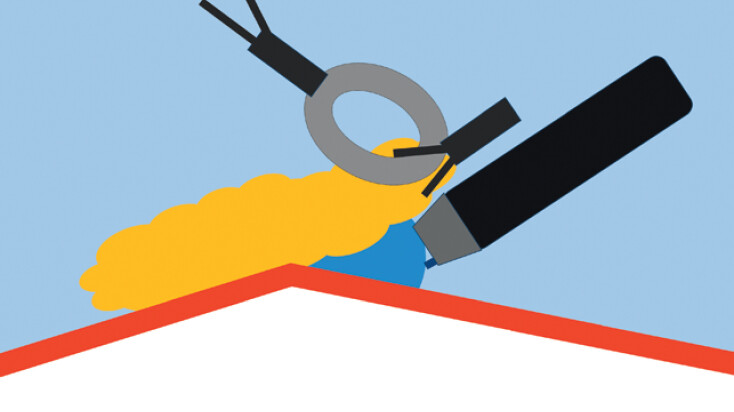
A lateral mucosal flap was created on the right side, and clip-and-band traction was applied to enhance exposure.

**Fig. 3:**
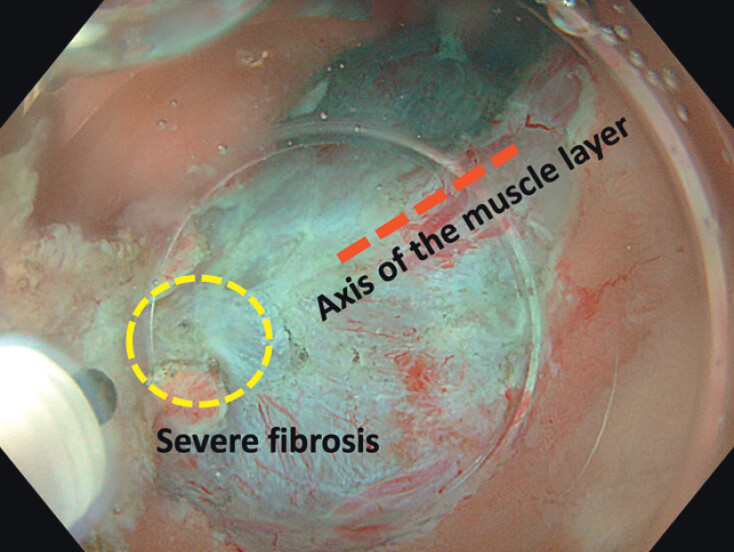
Severe fibrosis with the absence of a clear submucosal plane; further progression was unsafe due to tangential access.

Clip-and-band traction was applied to improve exposure and maintain tension on the dissection plane ( Fig. 2 ), as previously described. 3​ Submucosal dissection was performed using a water-jet knife connected to a pump, allowing continuous injection and precise cutting. The saline environment provided magnified visualization, minimized electrosurgical smoke, and enhanced the identification of submucosal fibers, which is particularly useful in fibrotic lesions. 2​ ​4​ In fibrotic areas, careful hook-and-drag movements were performed while maintaining a dissection plane parallel to the muscularis propria to reduce the risk of perforation ( Figs. 3 4 5 ).

**Fig. 4:**
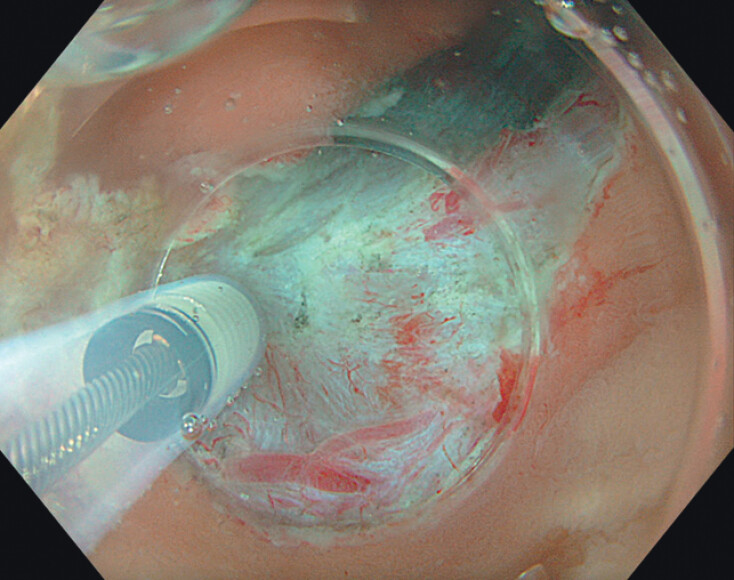
To improve access, the knife tip was inserted into the fibrotic segment and the fibers were gently hooked.

**Fig. 5:**
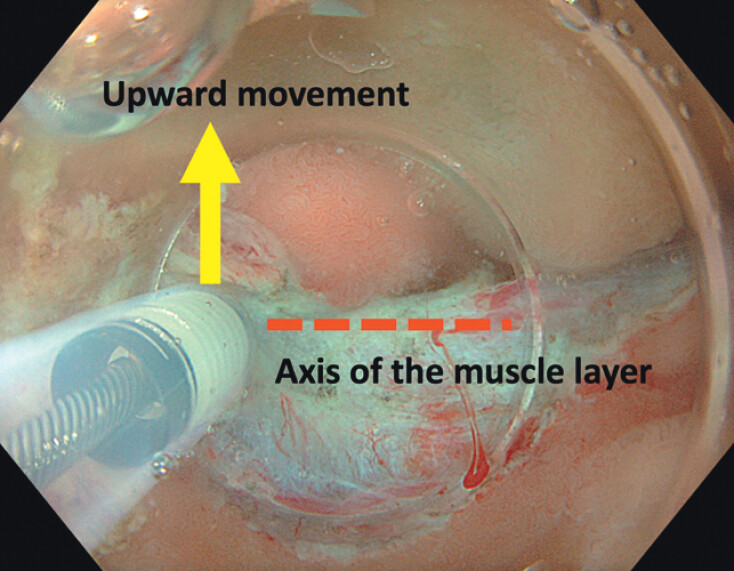
Gentle upward manipulation of the endoscope aligned the muscular layer parallel to the knife axis, facilitating safer dissection.

En bloc resection was achieved without intraprocedural or delayed adverse events. The resection site was completely closed using endoscopic clips, in accordance with current recommendations. 2​ The patient had an uneventful recovery and was discharged without complications. This combined approach may improve safety and efficacy in selected cases of duodenal ESD with significant fibrosis.

UCTN_Code_TTT_1AO_2AG_3AD
